# A Multimodal Deep Learning Approach to Predicting Systemic Diseases from Oral Conditions

**DOI:** 10.3390/diagnostics12123192

**Published:** 2022-12-16

**Authors:** Dan Zhao, Morteza Homayounfar, Zhe Zhen, Mei-Zhen Wu, Shuk Yin Yu, Kai-Hang Yiu, Varut Vardhanabhuti, George Pelekos, Lijian Jin, Mohamad Koohi-Moghadam

**Affiliations:** 1 Division of Periodontology & Implant Dentistry, Faculty of Dentistry, The University of Hong Kong, Hong Kong SAR, China; 2 Department of Implant Dentistry, Beijing Stomatological Hospital, Capital Medical University, Beijing 100050, China; 3 Division of Applied Oral Sciences & Community Dental Care, Faculty of Dentistry, The University of Hong Kong, Hong Kong SAR, China; 4 Division of Cardiology, Department of Medicine, The University of Hong Kong-Shenzhen Hospital, Shenzhen 518009, China; 5 Division of Cardiology, Department of Medicine, The University of Hong Kong, Queen Mary Hospital, Hong Kong SAR, China; 6 Department of Diagnostic Radiology, Li Ka Shing Faculty of Medicine, The University of Hong Kong, Hong Kong SAR, China

**Keywords:** periodontal disease, systemic comorbidity, multimodal deep learning, panoramic radiograph, electronic health records

## Abstract

**Background:** It is known that oral diseases such as periodontal (gum) disease are closely linked to various systemic diseases and disorders. Deep learning advances have the potential to make major contributions to healthcare, particularly in the domains that rely on medical imaging. Incorporating non-imaging information based on clinical and laboratory data may allow clinicians to make more comprehensive and accurate decisions. **Methods:** Here, we developed a multimodal deep learning method to predict systemic diseases and disorders from oral health conditions. A dual-loss autoencoder was used in the first phase to extract periodontal disease-related features from 1188 panoramic radiographs. Then, in the second phase, we fused the image features with the demographic data and clinical information taken from electronic health records (EHR) to predict systemic diseases. We used receiver operation characteristics (ROC) and accuracy to evaluate our model. The model was further validated by an unseen test dataset. **Findings:** According to our findings, the top three most accurately predicted chapters, in order, are the Chapters III, VI and IX. The results indicated that the proposed model could predict systemic diseases belonging to Chapters III, VI and IX, with AUC values of 0.92 (95% CI, 0.90–94), 0.87 (95% CI, 0.84–89) and 0.78 (95% CI, 0.75–81), respectively. To assess the robustness of the models, we performed the evaluation on the unseen test dataset for these chapters and the results showed an accuracy of 0.88, 0.82 and 0.72 for Chapters III, VI and IX, respectively. **Interpretation:** The present study shows that the combination of panoramic radiograph and clinical oral features could be considered to train a fusion deep learning model for predicting systemic diseases and disorders.

## 1. Introduction

Oral health, as an integral part of overall wellbeing, is an important indicator of general health and quality of life [1,2]. Periodontal (gum) disease, including gingivitis and periodontitis, is one of the major oral disease burdens worldwide, and it is characterized by dysregulated host immuno-inflammatory responses to oral dysbiotic biofilms, thereby resulting in destruction of tooth-supporting periodontal tissues [3]. Of note, a systematic review of the research activity in periodontal medicine reveals that 129 Medical Subject Headings (MeSH) terms representing 57 systemic diseases/disorders or comorbidities have been associated with periodontal disease [4]. Currently, to the best of our knowledge, there are no machine learning models attempting to identify systemic diseases on the basis of oral conditions. Here, we developed a multimodal deep learning method by fusing various features from panoramic radiographs (so-called orthopantomogram, OPG) and other oral clinical information taken from the electronic health record (EHR) to predict systemic diseases. 

In recent years, there has been a sharply rising interest in the advancement of artificial intelligence (AI) across all scientific disciplines. AI is a broad term that refers to a machine’s ability to learn and react in a similar way as humans do. AI-based systems are trained to perform a wide range of rational tasks without the need for specialized programming. These intelligent platforms are therefore commonly employed in real-world applications, such as image processing, text mining and speech recognition [5,6]. Furthermore, by incorporating AI into healthcare services, we could to some extent improve clinical diagnosis, treatment planning and development of new approaches, protocols or drugs [7]. Conceivably, AI-assisted diagnosis and treatment planning may become less expensive and more widely available in the near future. Indeed, machines can learn from large amounts of patients’ datasets to determine the fundamental characteristics of an individual patient, allowing clinicians to diagnose various diseases and disorders at an early stage for effective healthcare [8]. Notably, detection of brain tumors via MRI, retinopathy detection via eye images, cardiac health assessment via electrocardiograms and COVID epidemic management and analysis are some eye-catching examples of AI in healthcare [9,10,11].

Deep learning models have shown enormous promise in medical image processing for providing clinical decision-making support for a wide range of diseases and disorders such as diabetic retinopathy, cancer, and Alzheimer’s disease [12]. However, relying only on medical images is inadequate in clinical practice, and a more robust clinical decision could be made by incorporating various sources of data like structural laboratory results and medical history information from the EHR [13]. It has recently been demonstrated that using data fusion to train a multimodal deep learning model can enhance the accuracy and robustness of the final prediction [14]. In these models, features from medical images have been fused with other characteristics to improve the performance of the model. 

Medical images are usually high-dimension data with a large number of trainable features [15]. For instance, pixels in a CT image of the chest have sub-mm resolution, resulting in an imaging dataset containing a million or more voxels. Therefore, extracting the related features from the raw medical images would be a challenging step to build an efficient multimodal model. Using manual annotation to extract features is usually costly and time-consuming. This is especially true in the field of medical images, where the data are sensitive, and annotation requires a lot of specialized domain knowledge. 

Here, we used a two-phase approach to predict systemic diseases from oral condition. In the first phase we used a dual-loss autoencoder to extract periodontal disease-related features from 1188 oral radiographs of subjects with different periodontal conditions. The autoencoder does not require pixel-level annotation to extract the related features from the OPG radiographs. In the second phase, we fused these image features with clinical oral information taken from the EHR to train a deep neural network model for identifying the potential systemic comorbidities. Notably, the multimodal model trained by the fused features outperformed those only trained by one of such features. Our findings show that the proposed model could be used to predict systemic diseases based on oral conditions.

## 2. Materials and Methods

### 2.1. Dataset

The data were retrieved from the EHRs of Prince Philip Dental Hospital (PPDH) and Queen Mary Hospital (QMH). We collected age, gender, income, teeth number, periodontal stage, and bone loss (Table 1) for 1188 Chinese dentate adults (35–97 years with a median of 56 years; 61% of females). Mean imputation was used to replace missing values in 13 subjects where alveolar bone level was not measurable. The Digital Imaging and Communications in Medicine (DICOM) format of individual OPG file was collected from the PPDH database. All OPG images were assessed by a single examiner (DZ) and the subjects were then assigned to four groups based on the new classification of periodontal diseases and conditions [16]. We used OPG images along with the eight demographic and clinical features to train our model.

On the other hand, the medical records of patients were obtained from the EHR of QMH. The diseases/conditions were coded with the International Classification of Diseases (ICD)-10, and the disease profiles were then categorized into 14 Chapters (excluding K00-K14 which is “Diseases of oral cavity, salivary glands and jaws”) (Table S1) [17]. Next, all subjects were classified as two groups with the presence or absence of the diseases/conditions in each Chapter. This study was approved by the Institutional Review Board of the University of Hong Kong/Hospital Authority of Hong Kong West Cluster (IRB UW 16-434).

### 2.2. Dual-Loss Autoencoder to Extract Periodontal-Related Features from OPG

**Figure 1:**
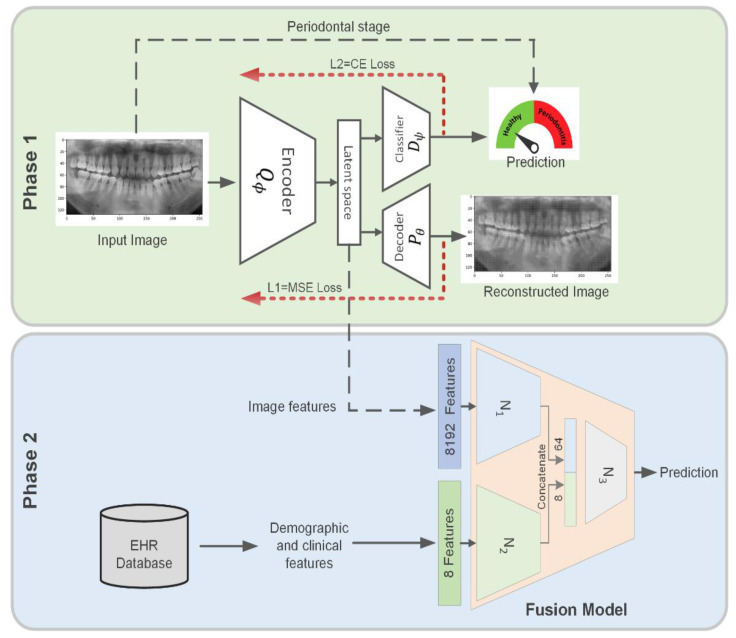
The overall schema of the model. Phase 1: We used the raw OPG images as input to an autoencoder to extract periodontal disease-related features. The autoencoder has a dual-loss architecture, with mean squared error (MSE) and cross-entropy (CE) losses. After training the autoencoder, the latent space of the model is used for the next step. Phase 2: The periodontal disease-related features (the latent space of the autoencoder) have been used as inputs in a deep neural network model. The patient demographic data extracted from the EHR were combined with the image features to train the model. Prediction of systemic diseases using the fusion model.

In the first phase, we extracted periodontal disease-related features from OPG images using a dual-loss autoencoder (Figure 1). To train the model, we used two losses: the mean squared error (MSE) loss [18] for input image reconstruction and the cross-entropy loss [19] for predicting patient periodontal stages. We labeled the patients into two groups including 544 (45.8%) in severe disease group (Stage III generalized and Stage IV Periodontitis) and 644 (54.2%) from the counterpart (periodontal health, gingivitis, Stages I-III Periodontitis, localized). 

For the given input image samples set $\left\{x^{\left(1\right)},x^{\left(2\right)},\ldots ,x^{\left(N\right)}\right\}$ where$\;x^{\left(i\right)}\in {\mathbb{R}}^{W\times L}$, the inputs are mapped by encoder $Q_{\phi }\left(x\right)$ to the latent representation of LR. The LR data propagate into two paths, one for reconstruction of the input image and another path for classification of images based on their periodontal stages. For the reconstruction path, the latent space fed into a decoder $P_{\theta }\left(LR\right)$ to reconstruct the same input images using MSE loss (Equation (1)).

(1) $$L_{1}=\sum_{i=1}^{N}E\left[{\left(x_{i}-P_{\theta }\left(Q_{\phi }\left(x_{i}\right)\;\right)\;\right)}^{2}\right]$$

Simultaneously, the LR array goes through a dense layer network of $D_{\psi }\left(LR\right)$ and is then classified using cross-entropy loss (Equation (2)).

(2) $$L_{2}=-\sum_{i=1}^{N}y_{i}\log\left(D_{\psi }\left(Q_{\phi }\left(x_{i}\right)\right)\right)$$

for OPG images we have a label set $\left\{y_{1},\;y_{2},y_{3},\;\ldots ,\;y\right\}\;$where$\;y_{i}\in \left\{0,1\right\}$. The final loss to train the autoencoder consists of these two losses:

(3) $$L_{LR}=L_{1,Q}+L_{2,D}$$

where the$\;\;L_{1,Q}$ is the backpropagated MSE loss through the decoder ($Q_{\phi }$) and the $L_{2,D}$ presents the loss of dense layers ($D_{\psi })$. 

Using two losses helps to avoid bias and makes the autoencoder robust against noise. The MSE loss could contribute to avoiding a possible bias in feature extraction by ignoring irrelevant information not essential to reconstruct the OPG image. In addition, applying cross-entropy loss assists to make a sparser latent space and leads to boosting the final fusion model to make it more robust against input noise, as the periodontal disease-related features could be distinguished in LR space. This would be helpful in OPG image processing, as the artifact of movement in imaging could be ignored by the sparsification of LR. 

We used a modified version of the BCDU-NET [20] for our image reconstruction model. For patient periodontal stages prediction, we used a fully connected network with three layers and Leaky-Relu activation function. The architecture of the model can be found in Figure S1. The training process was completed using Adam optimizer with a batch size of 32 in 10 epochs having 1e-4 learning rate and 10 extra epochs, with 1e-5 to be more precise. 

In the second phase, we fused the features extracted from the OPG images with the demographic and clinical oral features extracted from the EHR to train a deep neural network (DNN) (Figure 1). The DNN model is a binary classifier that uses dense and drop out layers to predict chronic diseases based on input features (Figure S2). As the number of features from the OPG images and the EHR is not the same (8192 and 8, respectively), we passed these two feature sets from the *N*_1_ and *N*_2_ networks prior to feature concatenation, making the number of features more equal and yet avoiding the potential bias in model training caused by a large number of OPG image features. Finally, we concatenated *N*_1_ and *N*_2_ output, and it was then fed into the *N*_3_ network for the final prediction (Figure 1).

For each ICD-10 chapter in our dataset, we trained a separated binary classifier model to predict if the patient belongs to that chapter or not. We used a down sampling approach to create a balanced dataset for those chapters so that the number of samples in the majority class was more than double the size of the minority class (extremely unbalanced). For each layer, the ReLU activation function was performed. For each layer L, we have:

$$u_{i}^{L}=ReLU\left[\overset{l-1}{\underset{p=0}{\sum }}W_{i}X_{i}+b^{L}\right]\;ReLU=\{\begin{array}{c} x,\;\;if\;x\geq 0\\ 0,\;\;if\;x<0 \end{array}\;$$

W denotes the weight matrix; X is the input, and b represents the bias value, and i is the indices of the output array *u.* Additionally, a drop out layer was utilized between each dense layer to minimize the overfitting of the model. Of note, we trained the model with a learning rate of 1e-4, batch size of 50, in 300 epochs. The architecture of the model can be found in the Figure S2.

## 3. Results

### 3.1. Extract Periodontal-Related Features from OPG

**Figure 2:**
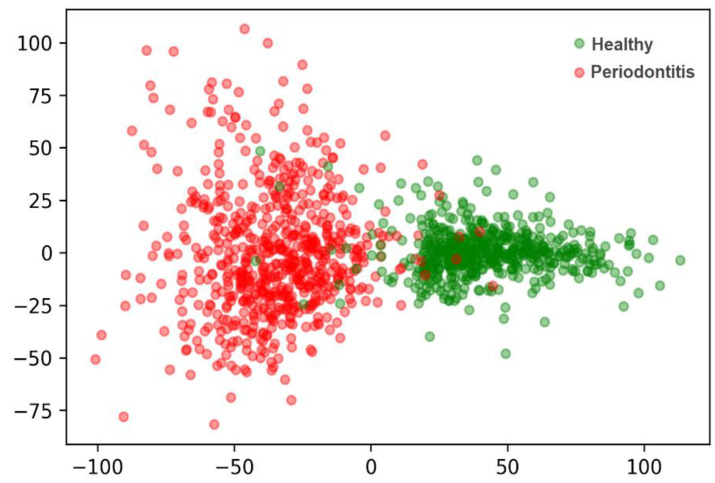
2D projection of the latent space of the autoencoder. The red dots show OPGs with periodontitis and the grey dots reflect healthy samples.

To assess the performance of the autoencoder model, a 2D projection of the latent space was employed. A principle component analysis (PCA) dimensionality reduction method was applied to the latent space of the OPG images. The plot shows two separate clusters formed by the healthy samples and OPG images with periodontitis (Figure 2). 

**Figure 3:**
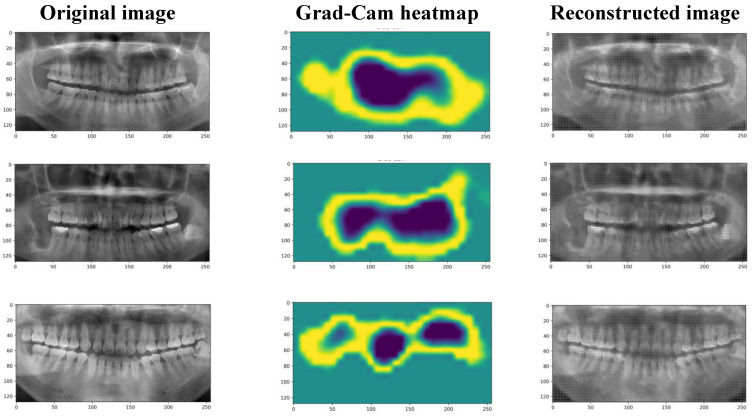
The reconstructed images by the autoencoder and the grad-cam heatmap. The brighter parts of the plots represent the areas that play a significant role in the model’s training phase.

Next, the discriminative regions were visualized via Grad-Class Activation Mapping (Grad-CAM) [21] for better realizing the regions that the network concentrates on while building the latent space. The activation maps help us determine which parts of the image make most of the contributions to the model’s final output. The model gave more attention to the brighter parts of the plots. These results revealed the autoencoder focused more on the periodontal area of the OPG images that was key for identifying periodontal disease. Figure 3 presents a graph of the Grad-CAM results for a group of OPG images.

### 3.2. Predict Systemic Disease Using a Fusion Model

**Figure 4:**
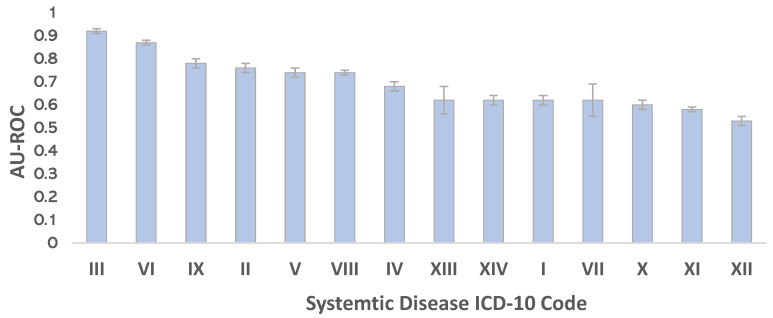
AU-ROCs of the 14 trained models. The results show the model has the best performance for the Chapters III, VI and IX.

In our dataset, we had systemic disease information for 14 ICD-10 chapters. Using the fused features, we trained 14 separate binary classifiers for each disease chapter to predict whether or not each patient belonged to that chapter. To evaluate the DNN models, the area under the receiver operator characteristic (ROC) curve was used. The data were divided between the training (70%) and unseen test (30%) for each disease chapter. For the training dataset, ten-fold cross-validation was undertaken, and the average of model performance in the ten-fold was presented. The results indicated that the model trained for Chapters III, VI and IX had the best performance among all 14 models, with AUCs of 0.92 (95% CI, 0.90–94), 0.87 (95% CI, 0.84–89) and 0.78 (95% CI, 0.75–81), respectively (Figure 4). 

**Figure 5:**
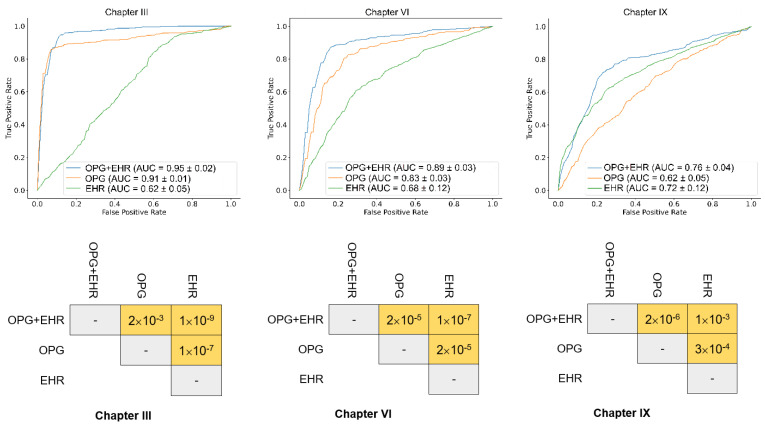
Ten-fold cross validation results for the training datasets (top row) and AUC comparison of the models using Delong test (bottom row). The model was trained with three different sets of features. Fused features of the OPG and clinical information showed the best performance.

To further determine whether combining OPG and EHR data features could improve the model performance, we trained models using OPG features or EHR data features alone, with reference to our fusion model, respectively. Moreover, the models were trained for predicting the top three disease chapters using only OPG image and EHR data features. The AUC results demonstrated that the model trained with the combined features outperformed those trained using only one type of the features. We also compared ROC curves using Delong’s paired AUC comparison tests (two-sided, null hypothesis = no difference in AUC) [22]. The *p*-values shows that models trained with both OPG and EHR features have a better performance (Figure 5).

**Figure 6:**
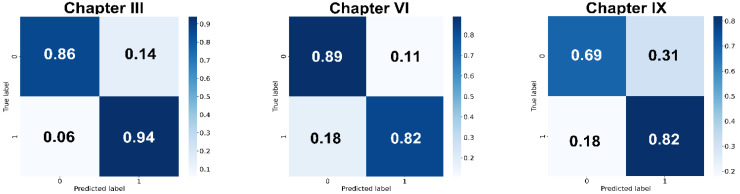
Normalized confusion matrix for the test unseen dataset.

Finally, to assess the robustness of the models, we performed the evaluation on the 30% of unseen dataset for Chapters III, VI and IX. The accuracy, sensitivity, specificity, precision and F1 score, and confusion matrix were used to evaluate our models on the unseen data. The results revealed the robustness of the model performance with an accuracy of 0.88, 0.82 and 0.72 for Chapters III, VI and IX, respectively (Table 2, Figure 6). 

In the present study, anaemia is the major disease identified in Chapter III. Chronic inflammation and persistent activation of the immune system may lead to anemia [23], likely due to insufficient production of erythropoietin and the decreased response of erythroid progenitors [24]. Consequently, the inhibition of hepcidin by erythroferrone is reduced, and the increased level of hepcidin alters the status of iron [25]. In addition, the release of iron stored in the body is also hindered by the persistent inflammation [26]. A recent systematic review indicated that periodontal disease, especially severe periodontitis, could reduce hemoglobin concentration and contribute to iron metabolism disorder [27]. Furthermore, as the leading cause of severe tooth loss in adults [28], periodontitis impacts nutritional intake, which may also account for anaemia. 

Among the subjects with conditions in Chapter VI, about 25% of them had sleep disorders (G47) and mononeuropathies of the upper limb (G56). According to the summary of 13 studies, the linkage of sleep disorders to periodontal disease exists, which may be induced by an elevated level of systemic inflammation in these patients [29]. Mononeuropathies of the upper limb could result in pain, weakness, and loss of upper extremity function, consequently limiting oral hygiene behaviors and increasing the risk of periodontal disease. In addition, Alzheimer’s disease (G30) is also coded in this Chapter. Recently, the keystone periodontopathogen *Porphyromonas gingivalis* and its gingipain have been detected in the brain specimens from patients with Alzheimer’s disease, which imply that periodontal disease may be involved in the pathogenesis of Alzheimer’s disease [30].

Furthermore, the diseases coded in Chapter IX have been widely documented, both epidemiologically and mechanically. In the present study, hypertension, ischaemic heart diseases, and stroke are the three most prevalent diseases from this Chapter with 349, 92 and 48 subjects, respectively. Indeed, periodontitis was proven to be an independent risk factor of atherosclerotic CVD in a large-scale cohort study with over 60 thousand participants [31]. Moreover, the landmark randomized controlled trial in periodontitis patients demonstrates that intensive periodontal treatment could improve endothelial function that would be potentially beneficial to enhance healthcare for CVD patients [32]. Our recent studies further show that periodontal therapy can favorably modulate the gene expression of inflammatory mediators in endothelial progenitor cells and yet contribute to improving the heart function in diabetic patients [33,34], as these promising treatment outcomes are connected to the underlying mechanisms of infection and inflammation. For example, the DNAs of periodontal pathogens are detectable from various endarterectomy specimens [35,36,37]. Indeed, these noxious pathogens and their virulence factors can crucially account for the endothelial dysfunction and the progression of CVD [38].

## 4. Discussion

As one of the most prevalent inflammatory diseases worldwide, periodontal disease is ranked 7th among all 369 diseases, injuries and impairments investigated in the extended Global Burden of Disease Study 2019 (GBD) [39]. Moreover, severe periodontitis is the most common cause of tooth loss and edentulism in adult population, and it substantially affects oral functions, nutritional intake and quality of life [28], with considerable socioeconomic impacts [40]. Notably, it is evident that periodontitis could crucially account for systemic dissemination of infections such as bacteremia, increased inflammation levels of the body and dysbiotic microbiomes in various niches (e.g., gut) [41]. Consequently, this serious oral inflammation is closely linked to various life-threatening noncommunicable diseases (NCDs), such as cardiovascular diseases (CVD), diabetes mellitus (DM), inflammatory bowel disease, respiratory diseases, Alzheimer’s disease (AD), chronic kidney disease, rheumatoid arthritis, pancreatic and colorectal cancers, various systemic comorbidities, and lately the COVID-19 [38,42,43,44,45,46,47,48]. In addition to these specific linking profiles of one systemic condition to periodontal disease, our 18-year follow-up study identifies for the first time that periodontitis experience may represent an increased risk for the onset of multiple common NCDs [49], and our latest findings further indicate that periodontitis is significantly linked to a cluster of systemic comorbidities [45].

In this study, we established a multimodal deep learning model to predict the presence of systemic diseases and disorders following the ICD-10 code, based on periodontal conditions assessed by alveolar bone levels and basic demographic characteristics. The benefit of our model is that it is trained using features from both medical images and patient clinical information to perform prediction. The dual loss autoencoder used in our model helps to identify the most powerful features from OPG radiographs without any pixel-level annotation. Additionally, we demonstrated that combining image and clinical features improves the model’s performance when compared to using only one type of feature. According to our findings, the top three most accurately predicted chapters in order are the Chapters III, VI and IX. 

While we demonstrated that deep learning models can be used to predict systemic diseases from oral conditions, there are some limitations to this approach that should be addressed. First, we were unable to find an appropriate external dataset on which to evaluate our model, due to the complexity of the combined dental and medical datasets. Second, our model could be expanded by including more data of clinical images and parameters to further improve the model’s performance for those systematic disease chapters where our current model has performed poorly. Further investigation would be highly warranted for screening and potentially identifying concurrent systemic diseases and conditions on the basis of oral/periodontal status, via applying the refined deep learning model. As such, it could be anticipated that the close collaboration and good teamwork of dental and medical professionals and computer science experts can better promote proactive disease prevention and deliver cost-effective personalized healthcare in the near future.

## 5. Conclusions

Within the limitations of the present study, various systemic diseases and disorders could be predicted according to oral conditions via the combination of panoramic radiographic findings and clinical oral/periodontal features by a fusion deep learning model. Oral/periodontal conditions may therefore be used conveniently for reflecting general health status and hopefully identifying concurrent systemic comorbidities in the future. It could be anticipated that medical professionals may well collaborate with dental and computer science experts for better promoting proactive disease prevention and thereby delivering precise and effective healthcare. Further evaluation and modification of this proposed model are highly warranted using the combined oral and medical datasets in different ethnic groups.

## Figures and Tables

**Table 1:** The eight demographic and clinical features.

EHR feature	Description
Age	Continuous
Gender	Binary
Income	Continuous
Number of teeth	Discrete
Periodontal stage	Continuous
Extent bone loss	Continuous
Bone loss max	Continuous
Age-adjusted bone loss	Continuous

**Table 2:** Performance of the models on the unseen test dataset.

Chapter Code	Accuracy	Sensitivity	Specificity	Precision	F1 Score
**III**	0.88 ± 0.01	0.93 ± 0.02	0.83 ± 0.03	0.85 ± 0.02	0.89 ± 0.01
**VI**	0.82 ± 0.02	0.84 ± 0.02	0.80 ± 0.06	0.81 ± 0.04	0.83 ± 0.01
**IX**	0.72 ± 0.02	0.77 ± 0.05	0.68 ± 0.04	0.71 ± 0.02	0.74 ± 0.03

## Data Availability

The raw OPG images and EHR data will be shared on reasonable request to the corresponding author. The code of the models is available in https://github.com/ClinicalAI/Fusion_DNN_OPG_EHR (accessed on 14 December2022).

## Supplementary Materials

The following supporting information can be downloaded at: https://www.mdpi.com/article/10.3390/diagnostics12123192/s1, Figure S1. The architecture of the dual-loss autoencoder; Figure S2. The architecture of the DNN model; Table S1. Chapters and their definitions in ICD-10.

## References

[1] Tonetti M.S., Bottenberg P., Conrads G., Eickholz P., Heasman P., Huysmans M.C., Lopez R., Madianos P., Müller F., Needleman I. (2017). Dental caries and periodontal diseases in the ageing population: Call to action to protect and enhance oral health and well-being as an essential component of healthy ageing—Consensus report of group 4 of the joint EFP/ORCA workshop on the boundaries between caries and periodontal diseases. J. Clin. Periodontol..

[2] World Health Organization (2022). Oral Health. https://www.who.int/health-topics/oral-health/#tab=tab_1.

[3] Page R.C., Kornman K.S. (1997). The pathogenesis of human periodontitis: An introduction. Periodontology 2000.

[4] Monsarrat P., Blaizot A., Kémoun P., Ravaud P., Nabet C., Sixou M., Vergnes J.-N. (2016). Clinical research activity in periodontal medicine: A systematic mapping of trial registers. J. Clin. Periodontol..

[5] Jain D.K., Liu X., Neelakandan S., Prakash M. (2022). Modeling of human action recognition using hyperparameter tuned deep learning model. J. Electron. Imaging.

[6] Zhang C., Lu Y. (2021). Study on artificial intelligence: The state of the art and future prospects. J. Ind. Inf. Integr..

[7] Liao K.-M., Liu C.-F., Chen C.-J., Shen Y.-T. (2021). Machine Learning Approaches for Predicting Acute Respiratory Failure, Ventilator Dependence, and Mortality in Chronic Obstructive Pulmonary Disease. Diagnostics.

[8] Aich S., Youn J., Chakraborty S., Pradhan P.M., Park J.-H., Park S., Park J. (2020). A Supervised Machine Learning Approach to Detect the On/Off State in Parkinson’s Disease Using Wearable Based Gait Signals. Diagnostics.

[9] Heidari A., Navimipour N.J., Unal M., Toumaj S. (2021). The COVID-19 epidemic analysis and diagnosis using deep learning: A systematic literature review and future directions. Comput. Biol. Med..

[10] Heidari A., Toumaj S., Navimipour N.J., Unal M. (2022). A privacy-aware method for COVID-19 detection in chest CT images using lightweight deep conventional neural network and blockchain. Comput. Biol. Med..

[11] Heidari A., Navimipour N.J., Unal M., Toumaj S. (2022). Machine learning applications for COVID-19 outbreak management. Neural Comput. Appl..

[12] Shen J., Zhang C.J.P., Jiang B., Chen J., Song J., Liu Z., He Z., Krittanawong C., Fang P.-H., Ming W.-K. (2019). Artificial Intelligence Versus Clinicians in Disease Diagnosis: Systematic Review. JMIR Public Health Surveill..

[13] Leslie A., Jones A.J., Goddard P.R. (2000). The influence of clinical information on the reporting of CT by radiologists. Br. J. Radiol..

[14] Huang S.-C., Pareek A., Seyyedi S., Banerjee I., Lungren M.P. (2020). Fusion of medical imaging and electronic health records using deep learning: A systematic review and implementation guidelines. NPJ Digit. Med..

[15] Berisha V., Krantsevich C., Hahn P.R., Hahn S., Dasarathy G., Turaga P., Liss J. (2021). Digital medicine and the curse of dimensionality. npj Digit. Med..

[16] Tonetti M.S., Greenwell H., Kornman K.S. (2018). Staging and grading of periodontitis: Framework and proposal of a new classification and case definition. J. Periodontol..

[17] https://icd.who.int/browse10/2016/en.

[18] Mathieu M., Couprie C., LeCun Y. (2015). Deep multi-scale video prediction beyond mean square error. arXiv.

[19] Zhang Z., Sabuncu M. (2018). Generalized cross entropy loss for training deep neural networks with noisy labels. Adv. Neural Inf. Process. Syst..

[20] Azad R., Asadi-Aghbolaghi M., Fathy M., Escalera S. Bi-directional ConvLSTM U-Net with densley connected convolutions. Proceedings of the 2019 IEEE/CVF International Conference on Computer Vision Workshop (ICCVW).

[21] Selvaraju R.R., Cogswell M., Das A., Vedantam R., Parikh D., Batra D. Grad-cam: Visual explanations from deep networks via gradient-based localization. Proceedings of the 2017 IEEE International Conference on Computer Vision.

[22] Delong E.R., Delong D.M., Clarke-Pearson D.L. (1988). Comparing the Areas under Two or More Correlated Receiver Operating Characteristic Curves: A Nonparametric Approach. Biometrics.

[23] Weiss G., Ganz T., Goodnough L.T. (2019). Anemia of inflammation. Blood J. Am. Soc. Hematol..

[24] Spoto B., Kakkar R., Lo L., Devalaraja M., Pizzini P., Torino C., Leonardis D., Cutrupi S., Tripepi G., Mallamaci F. (2019). Serum Erythroferrone Levels Associate with Mortality and Cardiovascular Events in Hemodialysis and in CKD Patients: A Two Cohorts Study. J. Clin. Med..

[25] Ganz T., Jung G., Naeim A., Ginzburg Y., Pakbaz Z., Walter P.B., Kautz L., Nemeth E. (2017). Immunoassay for human serum erythroferrone. Blood J. Am. Soc. Hematol..

[26] Schümann K., Solomons N.W. (2017). Perspective: What Makes It So Difficult to Mitigate Worldwide Anemia Prevalence?. Adv. Nutr. Int. Rev. J..

[27] Wu D., Lin Z., Zhang S., Cao F., Liang D., Zhou X. (2020). Decreased Hemoglobin Concentration and Iron Metabolism Disorder in Periodontitis: Systematic Review and Meta-Analysis. Front. Physiol..

[28] Tonetti M.S., Jepsen S., Jin L., Otomo-Corgel J. (2017). Impact of the global burden of periodontal diseases on health, nutrition and wellbeing of mankind: A call for global action. J. Clin. Periodontol..

[29] Schmidlin P.R., Khademi A., Fakheran O. (2020). Association between periodontal disease and non-apnea sleep disorder: A systematic review. Clin. Oral Investig..

[30] Dominy S.S., Lynch C., Ermini F., Benedyk M., Marczyk A., Konradi A., Nguyen M., Haditsch U., Raha D., Griffin C. (2019). *Porphyromonas gingivalis* in Alzheimer’s disease brains: Evidence for disease causation and treatment with small-molecule inhibitors. Sci. Adv..

[31] Beukers N.G.F.M., van der Heijden G.J.M.G., van Wijk A.J., Loos B.G. (2016). Periodontitis is an independent risk indicator for atherosclerotic cardiovascular diseases among 60 174 participants in a large dental school in the Netherlands. J. Epidemiol. Community Health.

[32] Tonetti M.S., D’Aiuto F., Nibali L., Donald A., Storry C., Parkar M., Suvan J., Hingorani A.D., Vallance P., Deanfield J. (2007). Treatment of Periodontitis and Endothelial Function. New Engl. J. Med..

[33] Wang Y., Liu H.N., Zhen Z., Yiu K.H., Tse H.F., Pelekos G., Tonetti M., Jin L. (2017). Periodontal treatment modulates gene expression of endothelial progenitor cells in diabetic patients. J. Clin. Periodontol..

[34] Wang Y., Liu H.N., Zhen Z., Pelekos G., Wu M.Z., Chen Y., Tonetti M., Tse H.F., Yiu K.H., Jin L. (2020). A randomized controlled trial of the effects of non-surgical periodontal therapy on cardiac function assessed by echocardiography in type 2 diabetic patients. J. Clin. Periodontol..

[35] Stelzel M., Conrads G., Pankuweit S., Maisch B., Vogt S., Moosdorf R., Flores-De-Jacoby L. (2002). Detection of *Porphyromonas gingivalis* DNA in Aortic Tissue by PCR. J. Periodontol..

[36] Zaremba M., Górska R., Suwalski P., Kowalski J. (2007). Evaluation of the Incidence of Periodontitis-Associated Bacteria in the Atherosclerotic Plaque of Coronary Blood Vessels. J. Periodontol..

[37] Figuero E., Lindahl C., Marin M.J., Renvert S., Herrera D., Ohlsson O., Wetterling T., Sanz M. (2014). Quantification of Periodontal Pathogens in Vascular, Blood, and Subgingival Samples From Patients With Peripheral Arterial Disease or Abdominal Aortic Aneurysms. J. Periodontol..

[38] Beck J., Papapanou P.N., Philips K., Offenbacher S. (2019). Periodontal Medicine: 100 Years of Progress. J. Dent. Res..

[39] GBD 2019 Diseases and Injuries Collaborators (2020). Global burden of 369 diseases and injuries in 204 countries and territories, 1990–2019: A systematic analysis for the Global Burden of Disease Study 2019. Lancet.

[40] Listl S., Galloway J., Mossey P., Marcenes W. (2015). Global Economic Impact of Dental Diseases. J. Dent. Res..

[41] Hajishengallis G., Chavakis T. (2021). Local and systemic mechanisms linking periodontal disease and inflammatory comorbidities. Nat. Rev. Immunol..

[42] Bui F.Q., Almeida-Da-Silva C.L.C., Huynh B., Trinh A., Liu J., Woodward J., Asadi H., Ojcius D.M. (2019). Association between periodontal pathogens and systemic disease. Biomed. J..

[43] Grossi S.G., Genco R.J. (1998). Periodontal Disease and Diabetes Mellitus: A Two-Way Relationship. Ann. Periodontol..

[44] Mattila K.J., Nieminen M.S., Valtonen V.V., Rasi V.P., Kesaniemi Y.A., Syrjala S.L., Jungell P.S., Isoluoma M., Hietaniemi K., Jokinen M.J. (1989). Association between dental health and acute myocardial infarction. BMJ.

[45] Zhao D., Wu M.-Z., Yu S.Y., Pelekos G., Yiu K.H., Jin L. (2022). Periodontitis links to concurrent systemic comorbidities among ‘self-perceived health’ individuals. J. Periodontal Res..

[46] Zhao D., Khawaja A., Jin L., Li K.Y., Tonetti M., Pelekos G. (2018). The directional and non-directional associations of periodontitis with chronic kidney disease: A systematic review and meta-analysis of observational studies. J. Periodontal Res..

[47] Wang Y., Deng H., Pan Y., Jin L., Hu R., Lu Y., Deng W., Sun W., Chen C., Shen X. (2021). Periodontal disease increases the host susceptibility to COVID-19 and its severity: A Mendelian randomization study. J. Transl. Med..

[48] Marouf N., Cai W., Said K.N., Daas H., Diab H., Chinta V.R., Hssain A.A., Nicolau B., Sanz M., Tamimi F. (2021). Association between periodontitis and severity of COVID-19 infection: A case–control study. J. Clin. Periodontol..

[49] Zhao D., Zhen Z., Pelekos G., Yiu K.H., Jin L. (2018). Periodontal disease increases the risk for onset of systemic comorbidities in dental hospital attendees: An 18-year retrospective cohort study. J. Periodontol..

