# Supplementary material for: A Multimodal Deep Learning Approach to Predicting Systemic Diseases from Oral Conditions
Source: Diagnostics (Basel). 2022 Dec 16;12(12):3192. doi: 10.3390/diagnostics12123192 (PMC9777898; doi:10.3390/diagnostics12123192)
Supplement: Supplementary file 1 [file diagnostics-12-03192-s001.zip › diagnostics-2088259-supplementary.pdf]

A multimodal deep learning approach to predicting systemic diseases from oral conditions

Supplementary information:

Table S1. Chapters and their definitions in ICD-10

Chapter	Block	Title
I	A00–B99	Certain infectious and parasitic diseases
II	C00–D48	Neoplasms
III	D50–D89	Diseases of the blood and blood-forming organs and certain disorders involving the immune mechanism
IV	E00–E90	Endocrine, nutritional and metabolic diseases
V	F00–F99	Mental and behavioural disorders
VI	G00–G99	Diseases of the nervous system
VII	H00–H59	Diseases of the eye and adnexa
VIII	H60–H95	Diseases of the ear and mastoid process
IX	I00–I99	Diseases of the circulatory system
X	J00–J99	Diseases of the respiratory system
XI	K00–K93	Diseases of the digestive system
XII	L00–L99	Diseases of the skin and subcutaneous tissue
XIII	M00–M99	Diseases of the musculoskeletal system and connective tissue
XIV	N00–N99	Diseases of the genitourinary system

Excluding K00-K14 (Diseases of oral cavity, salivary glands and jaws); Excluding N40-N99 (Diseases of male genital organs, Disorders of breast, Inflammatory diseases of female pelvic organs, Noninflammatory disorders of female genital tract, Other disorders of the genitourinary system).

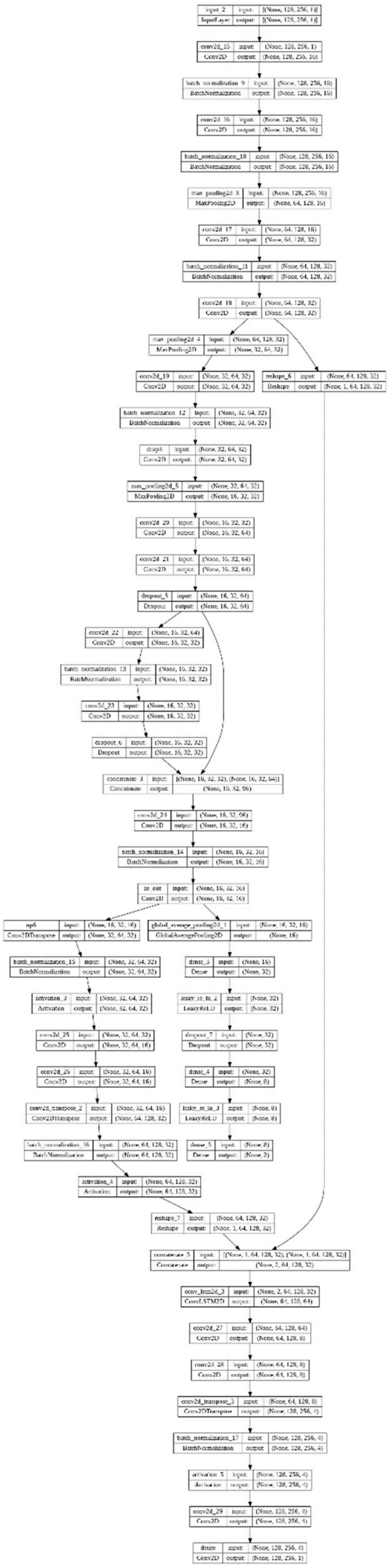

Figure S1. The architecture of the dual-loss autoencoder which we used in the phase 1 of the pipeline to extract the periodontal disease-related features from OPG images.

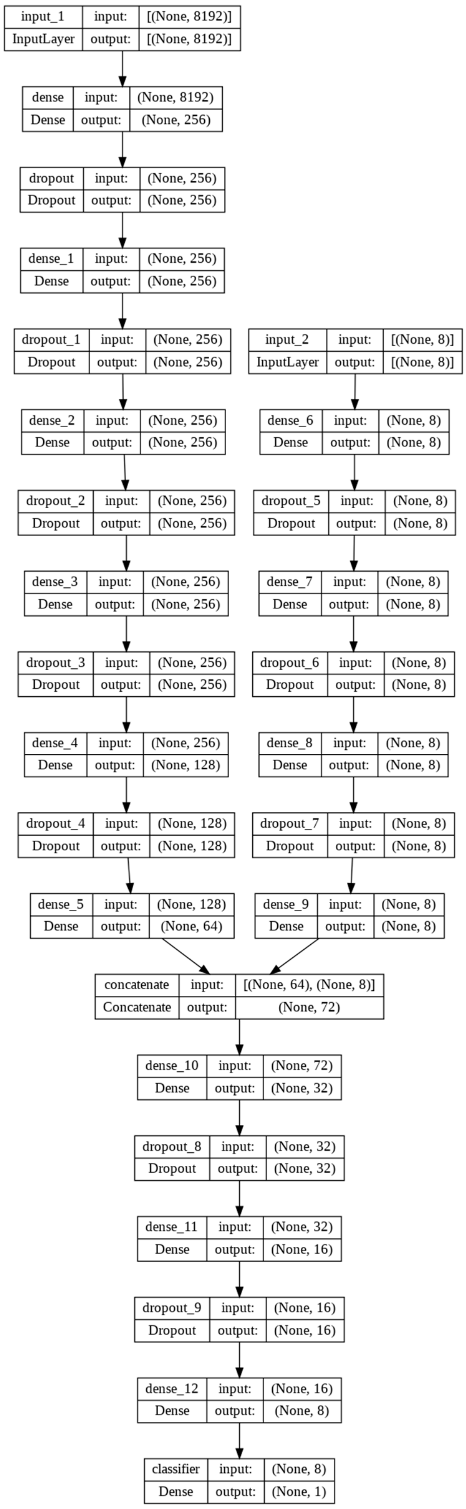

Figure S2. The architecture of the DNN model that we used in phase 2 of the pipeline to predict whether a patient has a systemic disease based on oral condition.
